# Bioactivity and In Silico Studies of Isoquinoline and Related Alkaloids as Promising Antiviral Agents: An Insight

**DOI:** 10.3390/biom13010017

**Published:** 2022-12-21

**Authors:** Divya Sharma, Neetika Sharma, Namish Manchanda, Satyendra K. Prasad, Prabodh Chander Sharma, Vijay Kumar Thakur, M. Mukhlesur Rahman, Mahaveer Dhobi

**Affiliations:** 1 School of Pharmaceutical Sciences, Delhi Pharmaceutical Sciences and Research University, Sector-III, Pushp Vihar, New Delhi 110017, India; 2 Delhi Institute of Pharmaceutical Sciences and Research, Delhi Pharmaceutical Sciences and Research University, Sector-III, Pushp Vihar, New Delhi 110017, India; 3 Department of Pharmaceutical Sciences, Rashtrasant Tukadoji Maharaj Nagpur University, Nagpur 440033, India; 4 Biorefining and Advanced Materials Research Centre, Scotland’s Rural College (SRUC), Kings Buildings, 11 West Mains Road, Edinburgh EH9 3JG, UK; 5 School of Engineering, University of Petroleum & Energy Studies (UPES), Dehradun 248007, India; 6 Pharmaceutical and Natural Products Chemistry, School of Health, Sports and Bioscience, University of East London, Stratford Campus, London E15 4LZ, UK

**Keywords:** isoquinoline and related alkaloid, BBI, anti-viral, SARS-CoV-2, COVID-19

## Abstract

Viruses are widely recognized as the primary cause of infectious diseases around the world. The ongoing global pandemic due to the emergence of SARS-CoV-2 further added fuel to the fire. The development of therapeutics becomes very difficult as viruses can mutate their genome to become more complex and resistant. Medicinal plants and phytocompounds could be alternative options. Isoquinoline and their related alkaloids are naturally occurring compounds that interfere with multiple pathways including nuclear factor-κB, mitogen-activated protein kinase/extracellular-signal-regulated kinase, and inhibition of Ca^2+^-mediated fusion. These pathways play a crucial role in viral replication. Thus, the major goal of this study is to comprehend the function of various isoquinoline and related alkaloids in viral infections by examining their potential mechanisms of action, structure-activity relationships (SAR), in silico (particularly for SARS-CoV-2), in vitro and in vivo studies. The current advancements in isoquinoline and related alkaloids as discussed in the present review could facilitate an in-depth understanding of their role in the drug discovery process.

## 1. Introduction

Human health has been impacted for decades by a variety of life-threatening viruses such as the hepatitis C virus (HCV), influenza virus, herpes simplex virus (HSV), hepatitis B virus (HBV), dengue virus (DENV), human immunodeficiency virus (HIV), human coronaviruses (HCoV), human cytomegalovirus (HCMV), and Zika virus. Viral infections have become a serious global concern for health workers due to their uncontrollable morbidity and death rate. The recent SARS-CoV-2 (severe acute respiratory syndrome-corona virus-2) has affected approximately 631 million people with 65 lakh deaths as per the WHO dashboard, on 14 November 2022. COVID-19 or SARS-CoV-2 is a novel coronavirus that shares 79 percent of its DNA with the SARS-CoV (severe acute respiratory syndrome-corona virus) and 50% of its DNA with the Middle East Respiratory Syndrome (MERS) virus [1,2,3]. Similarly, HIV is also a major health concern worldwide. In 2021, approximately 6 lakh deaths occurred due to HIV-related causes, and approx. 1.5 million people have been diagnosed with HIV viral infection [4]. It is estimated that at the end of 2021, people living with HIV infection reached up to 38.4 million. The global prevalence of HBV infection was 296 million in 2019 with 1.5 million new infections each year. Furthermore, it is reported that 2.7 million HBV-infected people are HIV-positive [5]. Due to the social behavior of humans, several viral diseases such the HIV, HBV, and HCV continue to have a significant negative impact on some geographical areas. Certain viral infections are also involved in the etiology of complex diseases such as hyperglycemia, Alzheimer’s disease, and cancer [6,7,8]. Various synthetic approaches are being developed by researchers including triazoles [9], peptides [10], and thiadiazole [11] to combat viral infections. However, the safest method to protect the community against viral infections is vaccination. However, due to viral mutation, vaccinations may frequently lose their effectiveness. Alternatively, they have not yet been developed; for example, in the Zika virus [12]. Recent mutating variants of COVID-19 such as alpha, beta, delta, and omicron also pose great challenges to the development of novel therapeutics [13]. There are currently no authorized treatments for several viruses, and immunization is only limited to the hepatitis A virus, mumps, and varicella [14]. Moreover, increased urbanization and worldwide travel have also resulted in pandemic outbreaks and ultimately made day-to-day life riskier. Thus, there is an urgent need to explore highly potent and cost-effective medicine to control newly emerged mutating variants of viruses.

Medicinal plants and their phytochemicals continue to play an important role in treating viral infection to inhibit virus entry, multiplication, and release [15,16,17]. Moreover, phytoconstituents also act as immune boosters to protect against viral infections [18,19]. Certain reports strongly suggest using drugs of natural origin in the treatment of complex viral infections including HIV and SARS-CoV-2 [20,21]. Recently, the role of phytochemicals in anti-inflammatory activity has also got considerable scientific attention for possible intervention in viral infections including SARS-CoV-2 [22,23]. Researchers are also exploring natural products to find the potentiality against newly emerged SARS-CoV-2 viral infection [24]. Numerous in silico, in vitro, and in vivo investigations are going on to find potential candidates against viral infections. The current review comprises in silico studies (particularly SARS-CoV-2) followed by in vitro and in vivo investigations of different categories of isoquinoline alkaloids and their related moieties against viral strains. The structure–activity relationship of a few significant subcategories of isoquinoline and related alkaloids are also included in this study. Some recent reports on isoquinoline alkaloids suggest their role in complex diseases such as Alzheimer’s, parkinsonism, cancer, and viral diseases [25,26].

## 2. Isoquinoline and Related Alkaloids: A New Ray of Hope

Alkaloids are natural organic compounds containing nitrogen atom/atoms in their heterocyclic ring and are mainly derived from amino acids. These basic nitrogenous compounds represent numerous medicinal properties such as anti-inflammatory [27,28], analgesic [29], antibacterial [30], anti-cancer [31], and anti-fungal [32]. For decades, the antiviral activity of naturally obtained alkaloids against different strains of viruses has been well documented [33,34,35].

**Figure 1:**
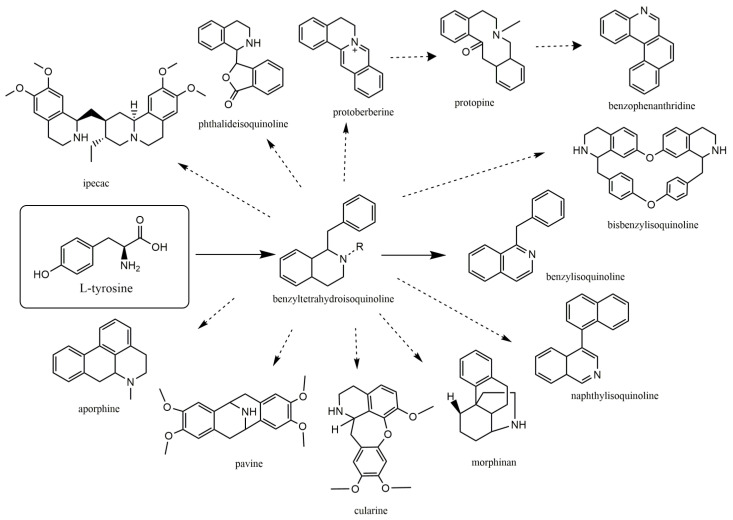
Some important L-tyrosine-derived isoquinoline and their related alkaloids.

**Figure 2:**
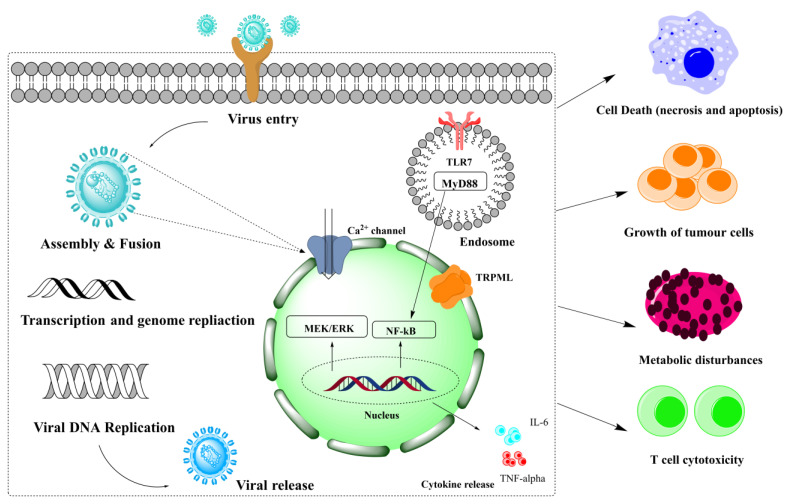
Various steps involved in the pathology of viral infections that might harm cells. MEK/ERK: Mitogen-activated protein kinase/extracellular-signal-regulated kinase, NF-κB: nuclear factor kappa B, TRPML: transient receptor potential, TLR7: Toll-like receptor (TLR)-7, MyD88: myeloid differentiation primary response protein, TNF-α: tumor necrosis factor alpha, IL-6: interleukin-6.

The second-largest category of alkaloids after indole alkaloids is isoquinoline alkaloids [36] which are mainly derived from L-tyrosine and phenylalanine. In the 19th century, morphine was the first isoquinoline alkaloid isolated from *Papaver somniferum* [37]. There is no structural homogeneity among the isoquinoline alkaloids [25,38,39]. Due to this reason, the classification of these alkaloids is a bit puzzling. However, based on varying levels of oxygenation, rearrangements at the intramolecular level, distribution, shared biosynthetic routes, and the presence of extra rings linked to the main moiety, isoquinoline alkaloids may be divided into 13 classes including benzyltetrahydro-isoquinoline, benzylisoquinolines, aporphines, protoberberine, benzophenanthridine, phthalideisoquinoline, morphinans, pavines, cularines, bisbenzylisoquinolines (BBI), naphythylisoquinoline, promorphinans, and ipecac alkaloids [26,39,40]. Interestingly, the primary precursor for biosynthesizing the other varieties of isoquinoline alkaloids in plants is the benzyltetrahydroisoquinoline type alkaloid (Figure 1). These alkaloids possess numerous biological activities including antifungal, antidiabetic, anticancer, anti-inflammatory, antibacterial, and antiviral. The possible mechanism by which isoquinoline-related alkaloids could show antiviral activity is illustrated in Figure 2 [26,40,41].

Recent research on viral infections has confirmed the potential role of various categories of isoquinoline and related alkaloids in the prevention of diseases such as SARS-CoV and SARS-CoV-2 [42,43,44].

## 3. Therapeutic Targets for SARS-CoV-2 Inhibition: In Silico Approaches

One of the main advantages of in silico drug designing process is its cost-effective nature in the research and development process. Molecular modeling and in silico methodologies have gained lots of attention nowadays. Thus, these approaches have been extremely useful in identifying targets and predicting the efficacy of new drugs in recent pandemics. In the recent SARS-CoV-2 infection cycle, angiotensin-converting enzyme (ACE2) fuses with SARS-CoV-2 spike protein to promote and facilitate the virus’s entry. Natural compounds inhibiting ACE2 will directly help in the management of COVID-19 as it restricts the entry of SARS-CoV-2 to the host cell. Similarly, inhibition of other target proteins such as spike protein, main proteases (M^pro^/3CL^pro^), RNA-dependent RNA polymerase (RdRp), and non-structural proteins (NS) may suppress the SARS-CoV-2 infection [45]. Isoquinoline and their related alkaloids effectively bind with all these above-mentioned targets of SARS-CoV-2 enlisted in Table 1.

### 3.1. Angiotensin-Converting Enzyme-2 (ACE2) and Spike(S) Protein

A docking study of 17 natural products including alkaloids such as thebaine and berberine (BBR) showed satisfactory binding with ACE2 and S-protein of SARS-CoV-2. Thebaine (−100.77 kcal/mol) and BBR (−97.54 kcal/mol) showed the strongest binding affinities toward ACE2. While binding with S-protein, thebaine interacted with Gln314, Arg765, and Thr768 residues demonstrating a binding score of −104.56 and −103.58 kcal/mol respectively, whereas, BBR showed interactions with Arg765, Asn317, and Ser316 with a binding score of −99.93 kcal/mol [46]. In one study, previously reported anti-coronavirus alkaloids were assessed through in silico study and a Libdock score was calculated. A BBI alkaloid cepharanthrine (CEP) was the second-highest alkaloid exhibiting a −106.74 kcal/mol binding score after homorringtonine. Other isoquinoline alkaloids such as lycorine (−86.92), tetrandrine (−72.96), and fangchinoline (−92.66) were effectively bound with the S1 subunit of SARS-CoV-2 [42].

### 3.2. Main Protease (M^pro^) or 3-Chemotrypsin-like Protease (3CL^pro^)

Apart from ACE2, the bis-benzylisoquinoline alkaloidal category shows potential binding affinity against M^pro^/3CL^pro^, PL^pro^, RNA-dependent RNA polymerase (RdRp), and other NS proteins. Berbamine and oxyacanthine are also bis-benzylisoquinoline alkaloids isolated from *Berberis asiatica* Roxb. ex DC. Docking of these compounds using AutoDock Vina demonstrated maximum binding affinity with the binding free energy of −20.79 kcal/mol and −33.35 kcal/mol against M^pro^ [48]. CEP obtained from *Stephania cepharantha* Hayata has been discovered to have a critical role in the prevention and management of COVID-19 [56]. This phytoconstituent and its natural analogues showed satisfactory results when evaluated collectively by in silico and in vitro methods. Moreover, tetrandrine was also assessed in this research but showed slightly lower activity than CEP [57]. CEP showed strong binding interaction against 3CL^pro^ (−8.5 kcal/mol) and TMPRSS-2 (−7.4 kcal/mol) when docked with the help of Swiss PDB viewer, PyRx, and PyMol software [52]. These studies indicate that bis-benzylisoquinoline alkaloids should be evaluated more for the prevention of SARS-CoV-2 infection.

An in silico and molecular dynamic investigation on three primary alkaloids namely BBR, choline, and tetrahydropalmatine from *Tinospora cordifolia* (Willd.) Miers was conducted with the help of Auto Dock and MG Tools of Auto DockVina software. BBR (−7.3 kcal/mole) had the highest affinity for the SARS-CoV-2 protein 3CL^pro^ [47]. Natural compound noscapine mainly present in the opium poppy plant also possesses high M^pro^ inhibition activity exhibiting a very high docking score (−292.42 kJ/mol) [49]. Further molecular dynamic (MD) simulation studies also confirmed the stability of noscapine with M^pro^ [58]. The M^pro^ inhibition of palmatine obtained from *T. cordifolia* was also performed with the help of the SwissDock server. The binding score of palmatine and other natural compounds such as gingerol, and BBR with SwissDock-based docking software EADock DSS showed greater than −8 kcal/mol binding affinity toward the M^pro^ [50]. Molecular docking of a bis-benzylisoquinoline alkaloid oxycanthine showed a binding affinity of −10.99 kcal/mol via Auto Dock which ultimately suggested that it could be a successful candidate in the treatment or prevention of SARS-CoV-2. BBR and capsaicin also showed slightly good binding energy (−7.910 kcal/mol and −5.510 kcal/mol respectively) than caffeine [59]. A recent in silico study, using Autodock (version 4.2) on selected isoquinoline alkaloids including cephaeline, coptisine, galanthamine, glaucine, drotaverine, chelidonine, hydrastine, boldine, fumaricin stated that coptisine had the best binding affinity (−9.15 kcal/mol) toward M^pro^. MD simulation study of coptisine depicted a stable complex of N3-M^pro^ with a binding energy of −8.17 kcal/mol [54].

### 3.3. RNA-Dependent RNA Polymerase (RdRp)

RdRp is an important target to cure RNA-based viral diseases such as MERS, SARS, and SARS-CoV-2 as it is responsible for viral RNA replication. In SARS-CoV-2 infection, RNA genome replication is a prominent step to spread the infection; however, RdRp inhibitors prevent this step and treat the infection. Molecular docking of 38 Chinese patent drugs including phytocompounds, flavonoids and alkaloids, was studied for a molecular docking study against RdRp, ACE2, and M^pro^. The in silico study, with the help of AutoDock Vina, the findings demonstrated that morphine, codeine, indirubin, and BBR showed effective binding capacity (>−6.0 kcal/mol) against M^pro^; however, their binding affinity against RdRp was much higher (>−8.1 kcal/mol) than that of M^pro^ [51]. CEP also showed binding with RdRp protein to inhibit viral replication [43]. A study includes 143 isoquinoline compounds for in silico screening using Molegro Virtual Docker (version 6.0, Molegro ApS, Aarhus, Denmark) against RdRp of the Zika virus. Molecular docking studies also demonstrated that Cassiarin D possessed a high binding score against RdRp (−150.7 kJ/mol) [53].

## 4. Antiviral Actions of Isoquinoline Related Alkaloids

Natural isoquinoline and its related alkaloids have been widely explored in recent years for their antiviral potential. For instance, BBR is a protoberberine alkaloid extracted from various species of the *Berberis* genus exerting antiviral activity against a diverse range of viruses including HIV, HSV, HCMV, and human papillomavirus (HPV) [27]. BBR interferes with certain pathways such as nuclear factor-kappa B (NF-κB), mitogen-activated protein kinase/extracellular-signal-regulated kinase (MEK/ERK), and mammalian target of rapamycin and the adenosine monophosphate-activated protein kinase (AMPK/mTOR) to resist viral replication, thereby, providing antiviral action. Other antiviral activities of isoquinoline and related alkaloids are summarized in Table 2.

### 4.1. Protoberberine Alkaloids

Protoberberine alkaloids contain a tetracycline ring system and are mainly derived from the benzyltetrahydroisoquinoline by oxidation of the phenolic group and coupling with the N-methyl group of isoquinoline [36]. Natural protoberberine alkaloids have multiple pharmacological actions such as antiseptic, sedative, stomachic, analgesic action, and antiviral action [112].

Protoberberine alkaloids show prominent effects against HSV, HBV, SARS-CoV, SARS-CoV-2, and HCMV. A protoberberine alkaloid berberine (BBR) isolated from various species of *Berberis* represents antiviral against HIV by inhibiting tumor necrosis factor (TNF-α) and IL-6 in macrophage of cultured mouse J774A [61]. Inhibition of the NF-κB pathway and c-Jun N-terminal kinase (JNK) phosphorylation is the main mechanism behind the anti-HSV action of BBR [60]. A complex of ZnO/BBR complex was also tested against SARS-CoV-2 by performing a plaque reduction assay and Vero E6 cell anti-viral assay. Molecular docking of this complex indicates the potential to inhibit papain-like protease (PL^pro^), spike protein, and spike-receptor-binding domain (RBD). Further, an in vitro study concludes that ZnO/BBR at various concentrations showed PL^pro^ inhibition in the range of 70–98% and thus, suggested its role in SARS-CoV-2 infection. The complex inhibits S protein-ACE2 binding as compared to single BBR and ZnO-NP [64]. A review also showed the antiviral potential of BBR in different strains of coronaviruses [27].

Recently, a nanomedicine containing BBR (NIT-X) was investigated against SARS-CoV and SARS-CoV-2. This nano-oral formulation increased CD8^+^ cells and interferon-gamma (IFN-γ), thus showing immunomodulatory activity [62]. A study on Vero E6 cells evaluated the effectiveness of BBR and chloroquine along with synthetic drugs like lopinavir, remdesivir, and cyclosporine. IC_50_ values of chloroquine (1.38 μM) and BBR (10.58 μM) suggested their anti-SARS-CoV-2 activity [63]. Moreover, in vitro studies on calu-3 cells demonstrated the potential of this nanoformulation to inhibit ACE2, TMPRSS2, IL-1α, IL-8, and IL-6 [62]. Furthermore, inhibiting pro-inflammatory mediators and improving immunomodulation would be an excellent therapy against SARS-CoV-2. A recent study included BBR and obatoclox for the assessment of anti-SARS-CoV-2 activity in Vero E6 cells and nasal epithelial cells, which showed EC_50_ values of BBR is 9.1 µM, thus confirming the role of BBR in reducing the viral replication that justifies its promising effects against SARS-CoV-2 [65]. BBR was also found to be effective against severe post-COVID conditions such as pulmonary fibrosis and reduced inflammation during COVID-19 pneumonia [113]. The in vivo study of BBR shows activity against influenza viral pneumonia. It can cause reduction in pulmonary edema and lung index in mice and ultimately suppress lung hemorrhage [114]. BBR also showed effectiveness against chikungunya virus through decreasing viral load in wild-type C57BL6/J mice. This isoquinoline alkaloid significantly reduced joint swelling in mice [67]. Moreover, BBR also shows another anti-influenza activity by decreasing viral titers in the mouse lungs and thus eventually cause reduction in the mortality rate of mice [115].

Other protoberberine alkaloids such as dehydrocavidine, BBR, dehydroapocavidine, dehydroisoapocavidine, and dehydroisocorypalmine were derived from *Corydalis saxicola* Bunting plant and proved to possess anti-HBV activity. The in vitro assay performed on the 2.2.15 cell lines showed that dehydrocavidine and dehydroapocavidine represent 51% and 54% inhibition against HBeAg respectively [71,116]. Similarly, a study performed on the isoquinoline alkaloids namely saxicolalines A and N-methylnarceimicine and other alkaloids of *C. saxicola* showed anti-HBV activity. Along with these two isoquinoline alkaloids, dihydrochelerythzrine (IC_50_ < 0.05 µM) also demonstrated potent activity against HBV [71]. An anti-HBV study conducted on 18 isoquinoline alkaloids obtained from the various plant species also demonstrated potent in vitro activity against HepG2.2.15 cell lines [117]. A few alkaloids such as columbamine iodide and jatorrhizine chloride also showed activity against HIV with IC_50_ values of 58 and 71 µg/mL respectively [118].

BBR can inhibit Toll-like receptor-7 (TLR7), NF-κB, and myeloid differentiation primary response 88 (MyD88) in the TLR7 signaling pathway and thus restrict viral copies in mice, which is attributed to the treatment of influenza viral infection (H1N1) [69]. A study indicates that BBR can inhibit DNA polymerase and immediate-early (IE-3) proteins. BBR has also been evaluated against different strains of HCMV and exerted broad-spectrum anti- HCMV activity due to its suppressive nature against murine CMV (MCMV) showing EC_50_ value within the range of 1.30 to 2.70 µM [70]. Another study performed on cell lines indicated that BBR suppresses viral replication of influenza A virus by interfering the MEK/ERK pathway. Different cell lines studies of BBR including human alveolar basal epithelial cell (A549), lung epithelial type 1 (LET1), primary human airway epithelial cells (HAE), and Madin–Darby canine kidney (MDCK) represented IC_50_ values of 17 µM, 4 µM, 16 µM, and 52 µM respectively [68]. BBR is able to suppress IL-6, IL-1β, IL-1α, and TNF-α by inhibiting Nod-like receptor protein 3 (NLRP-3) pathway [119]. 

**Figure 3:**
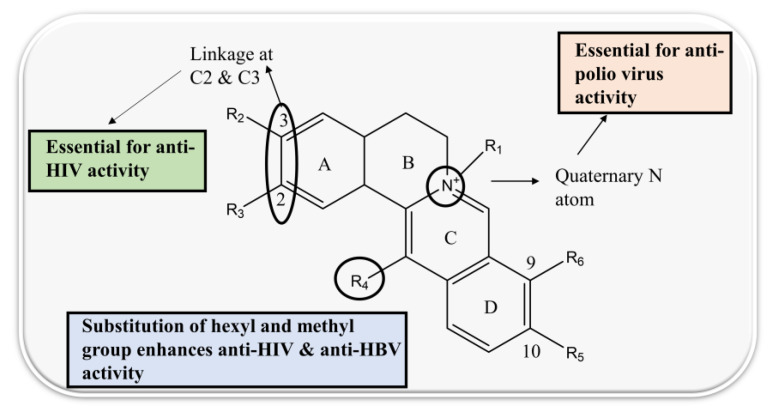
Structure–activity relationship of protoberberine alkaloids.

The structure–activity relationship of protoberberine alkaloids indicates that linkage between C2 and C3, a quaternary nitrogen atom and substitution with hexyl and methyl is important for anti-HIV, anti-HBV, and anti-polio activities [26,71,116,120,121] (Figure 3).

### 4.2. Aporphine Alkaloids

Aporphine alkaloids have been known for their antioxidant, anticancer, anthelmintic, antibacterial, antimalarial, and antiviral activity [122]. The in vitro activities of these alkaloids show potential action against HIV, HCV, and HSV viruses. A study of ixoratannin A-2 and aporphine alkaloid boldine on CEM-GXR cells showed anti-HIV activity with EC_50_ values of 34.4 and 50.2 µM. This in vitro study demonstrated the anti-HCV effect of ixoratannin A-2 and boldine [73]. Similarly, another category such as aporphine alkaloids also exerted anti-HIV action. For example, twelve compounds were isolated from the plant *Dasymaschalon rostratum* Merr. and Chun from which raymarine A, 3-methoxyoxoputerine-N-oxide, and dasymaroine B showed EC_50_ values in the range of 1.93 to 9.70 µM and exerted potent activity against HIV [75]. Moreover, marine alkaloids such as lamellarins alkaloids also exhibited anti-HIV action [123,124]. A natural steroidal alkaloid obtained from marine sponge didehydro-cortistatin has shown its potential against HIV-mediated inflammation [125]. An antiviral study included didehydro-cortistatin and highlighted ‘‘Block-and-lock’’ approach for the treatment of HIV. Didehydro-cortistatin A causes a reduction in HIV transcription and suppression of viral rebound in bone marrow-liver-thymus mice [126].

**Figure 4:**
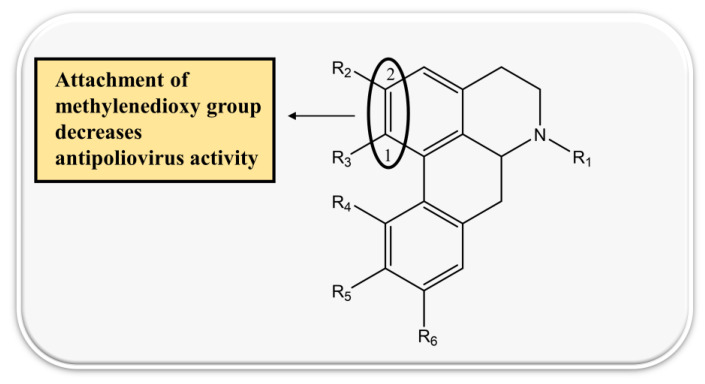
Structure–activity relationship of aporphine alkaloids.

Two aporphine alkaloids namely magnoflorine and langinosine from the plant *Magnolia grandiflora* L. possessed strong HIV inhibition potential. Moreover, further cytotoxic assays were carried out using tumor cell lines to determine anti-HSV activity. The results showed anti-HSV action of *M. lanuginosine* methanolic extract with 76.7% inhibition and 47% inhibition against polio-virus. Both the alkaloids magnoflorine and lanuginosine represent potent cytotoxic activity also with IC_50_ values of 0.4 and 2.5 µg/mL [127]. Nineteen aporphine alkaloids were also investigated on Vero cells of monkey kidneys against HSV, and results indicated the selection of three alkaloids namely oliverine HCl, pachystaudine, and oxostephanine as potent inhibitors of HSV [77]. The structure–activity relationship of aporphine alkaloids is demonstrated in Figure 4.

### 4.3. Benzyltetrahydroisoquinoline and Benzylisoquinoline

**Figure 5:**
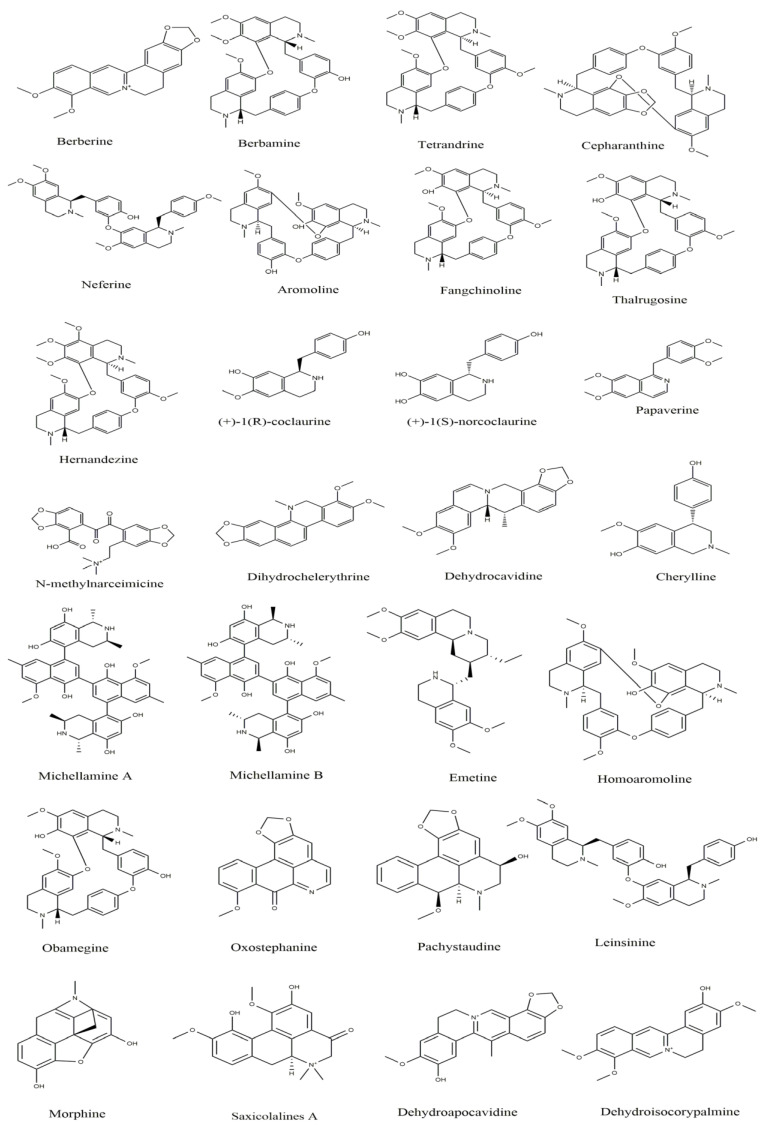
Chemical structures of isoquinoline and related alkaloids falling under various categories.

These type II isoquinoline alkaloids contain a three-ring system in which the B ring is reduced at C1-C2 and C3-C4. These alkaloids are the main precursor for the biosynthesis of many naturally occurring alkaloids including BBI, protoberberine, protopine, phthalideisoquinoline, and many others [36,128]. The chemical structures of several isoquinoline related alkaloids are given in Figure 5. An anti-viral study of 22 traditional medicinal plants including *Cimicifuga racemosa* (L.) Nutt, *Coptidis rhizoma, Meliae cortex, Phellodendron cortex,* and *Sophora subprostrata* Chun and T. Chen was found to be effective against coronavirus activity [129]. Major phytocompounds such as tylophorine, reserpine, lycorine, myricetin, scutellarin, apigenin, luteolin, emetine, emodin, aescin, and betulonic acid possess antiviral action, particularly against SARS-CoV [130,131,132,133,134,135]. Lycorine and emetine showed in vivo antiviral activity against HCoV-OC43 and MERS-CoV in mice model [111].

Two isoquinoline alkaloids namely (+)-1(R)-coclaurine and (+)-1(S)-norcoclaurine from *Nelumbo nucifera* Gaertn showed EC_50_ values of 0.8 and >0.8 µg/mL respectively against HIV [72]. Synthetic tetrahydroisoquinolines alkaloids were also developed and evaluated for antiviral activity in this study. The in vitro assay showed that a non-halogenated isoquinoline compound ethyl-1-benzyl-7-methoxyisoquinoline-4-carboxylate (6d) showed potent inhibitory activity against HIV [136].

The in vitro examination of papaverine hydrochloride against HIV with the help of human T-cell line such as MT4 cells showed effective ED_50_ values of 5.8 µM at a dose of 30 µM [79]. A similar in vitro study conducted on papaverine using H9 cells in peripheral blood mononuclear cell (PBMC) culture indicated potent antiviral activity against HIV at a concentration of 10 µg/mL [137]. The anti-HIV activity of salsolinol, tetrahydropapaveroline, and N, N-dimethylsalsolinol was performed on Raji cells. These three isoquinoline alkaloids also showed activity against Epstein-Barr virus early antigen (EBV-EA) [138]. Dimethoxy-3,4-dihydro isoquinoline and dihydroxyisoquinolinium salts were also reported effective against HIV with IC_50_ values of 2.07 µg/mL and 23.6 µg/mL respectively. EC_50_ values of both the compounds were found to be >0.10 µg/mL [139]. Thirty-three isoquinoline alkaloids selected from *Corydalis* and *Fumaria* species represented antiviral activity against herpes simplex and para-influenza virus. The in vitro study of alkaloids against anti-HSV and anti-PI3 activity using Vero and madine-darby bovine kidney (MDBK) cell lines represents effective results [76].

### 4.4. Bisbenzylisoquinoline (BBI)

BBI comprises two benzyltetrahydroisoquinoline that is combined via phenolic oxidation to form bisbenzylisoquinoline [36]. However, the main precursor for the BBI alkaloids is tyrosine. These alkaloids are known to have antibacterial, antifungal, antiviral, and antimalarial activity [140].

This famous subcategory of isoquinoline alkaloids shows a potential role in viral infections. In a study, methanolic extract from Indonesian plants was investigated against HSV-1. Later the same researcher evaluated subtypes of isoquinoline alkaloids obtained from water and methanolic extract of Chinese medicinal plants by subjecting them to plaque reduction assay to investigate the anti-HSV activity. All BBI alkaloids included in the study were found to be effective and showed IC_50_ values in the range of 14.8–43.2 µg/mL. Results confirmed that homoarmoline, isotetrandrine, berbamine, thalrugosine, and obamegine demonstrated anti-HSV-1 and HSV-2 potential with IC_50_ in the range of 16.3 to 24.9 µg/mL [93,102].

CEP, a plant alkaloid found in *Stephania cepharantha* from the family Menispermaeceae showed antiviral activity against HIV in U1 cells through inhibition of the NF-κB pathway [94]. A study revealed another mechanism of CEP against HIV by decreasing plasma membrane fluidity [96]. CEP also inhibits pro-inflammatory mediators to prove its anti-inflammatory action [95,99]. The synthetic analog of CEP demonstrated effectiveness against HIV-1 [99]. Two alkaloids from the plant *S. cepharantha* roots namely aromoline and di-O-acetylsinococuline indicate HIV inhibitory activity with CC_0_ values of 62.5 and 15.6 µg/mL, and IC_100_ value of 31.3 and 7.8 µg/mL [80]. Synthetic derivatives of cepharanoline were investigated against HIV in U1 cells. Out of all the analogues of cepharanoline, five compounds were selected and showed more potent inhibition than CEP [97]. Furthermore, CEP in combination with the 8-difluoromethoxy-1-ethyl-6-fluoro-1,4-dihydro-7-[4-(2-methoxyphenyl)-1-piperazinyl]-4-oxoquinoline-3-carboxylic acid (K-12) also reduced viral infection by inhibiting NF-κB and TNF-α in U1 cells [141]. In vitro activity of CEP showed potent activity against HSV also with an IC_50_ value of 0.835 µg/mL [142]. The antiviral activity of some important alkaloids including tetrandrine, CEP, neferine, and hernandezine on HEK 293T cells expressing ACE2 (293T-ACE2 cells) exhibited half-maximal effective concentrations (EC_50_) less than 10 µM [85], indicating inhibition of viral entry against SARS-CoV-2. Another in vitro assay was performed to identify CEP and nelfinavir (NFV) as anti-viral agents. N-protein expression and RNA levels were significantly decreased by NFV and CEP which suggested them to be potent drugs for COVID-19 [101].

It has been reported that CEP possessed anti-inflammatory and immunomodulatory action by suppressing cytokine release, NF-κB, nitric oxide (NO), and cyclooxygenase (COX). A newly established cell-culture model GX-P2V (pangolin coronavirus) such as SARS-CoV-2 model is being used to identify therapeutic drugs against COVID-19. CEP has been evaluated by this novel cell culture model and showed the highest inhibition against GX-P2V with an EC_50_ value of 0.98 µmol/L. In addition, CEP also inhibited viral entry and multiplication in the host cell and exerted maximum antiviral activity [143]. Similar research performed on the same cell culture model along with transcriptome analysis showed the potential of CEP in inhibiting viruses through various pathways including cellular stress responses and autophagy [144]. Thus, considering the above facts, CEP could be beneficial for treating COVID-19. The in vivo study of CEP demonstrated inhibition of porcine epidemic diarrhea virus (PEDV) through reduction in viral load at a dose of 11.1 mg/kg of b.w. [145]. Another bisbenzylisoquinoline alkaloid dauricine, isolated from *Menispermum dauricum* in combination with clindamycin could inhibit NF-κB activation in mice to treat influenza viral H5N1 infection [146].

Two bis-benzyl isoquinoline alkaloids including tetrandrine and isotetrandrine isolated from the roots of *Mahonia bealei* (Fortune) Pynaert possessed anti-influenza activity at a concentration of 0.25 mg/mL [147]. Tetrandrine (30 mg/kg, i.p.) in combination with acyclovir (120 mg/kg i.p.) proved to have anti-HSV activity in the Bragg albino mouse model (BALB). This BBI alkaloid was found responsible to inhibit IL-6 in the cornea of mice and thus exerts inhibition against HSV [84]. It was observed that tetrandrine suppressed delayed-type hypersensitivity and produces inflammatory responses to the virus [148]. In addition to tetrandrine two other alkaloids including fangchinoline, and CEP were investigated against human coronavirus OC43 (HCoV-OC43) using a Medical Research Council cell strain 5 (MRC-5) fibroblast lung cell line. Results demonstrated dose-dependent inhibition of tetrandrine, fangchinoline and CEP with IC_50_ values of 0.33, 1.01, and 0.83 µM respectively [83].

BBI alkaloid neferine was also assessed for anti-SARS-CoV-2 activity along with other 29 compounds. huh7, HEK293/hACE2 cell lines were used to check the potential of molecules toward SARS-CoV-2. This bis-benzylisoquinoline alkaloid possessed significant inhibition (approx. 75%) against SARS-CoV-2 pseudovirus and showed an EC_50_ value of 0.36 µM. It blocked the viral entry by inhibiting Ca^2+^-mediated fusion and thus could be a promising agent to treat viral infections including newly emerged SARS-SoV-2 [88]. Cycleanine, a BBI alkaloid, possessed potent anti-HIV efficacy with an EC_50_ value of 1.83 µg/mL [81]. Another BBI alkaloid, fangchinoline, also showed its effectiveness against various strains of HIV, showing EC_50_ value in the range of 0.8 to 1.7 µM [82].

Berbamine, isolated from *Berberis amurensis* Rupr., demonstrated its anti-COVID-19 activity by causing a reduction in ACE2 and dipeptidyl peptidase-4 (DPP-IV) levels in the plasma membrane. A study conducted on Huh7-cells indicates that berbamine caused inhibition of Ca^2+^ influx, which ultimately led to the inhibition of TRPMLs, which later resulted in the intervention of virus entry [91]. Moreover, berbamine also showed inhibition of the 2-E channel with an IC_50_ value of 111.50 µM, thus preventing SARS-CoV-2 infection. The EC_50_ value of this BBI alkaloid confirmed its inhibitory action against other flaviviruses as well as Japanese encephalitis virus (JEV) (1.62 mM) and Zika virus (2.17 mM) [91]. The β-carboline and isoquinoline alkaloids such as dictamine, cinchonine, and skimmianine showed inhibition against SARS-CoV. The quinine derivative, chloroquine also showed potent inhibition toward SARS-CoV-2 [149]. Multiple compounds were studied in vitro to investigate action against SARS-CoV-2 on the human epithelial colorectal adenocarcinoma cell line (Caco-2 cells). Top hits included synthetic drugs as well as phytocompound present in plants namely lopinavir, camostat, nafamostat, mefloquine, papaverine, and cetylpridinium [78].

**Figure 6:**
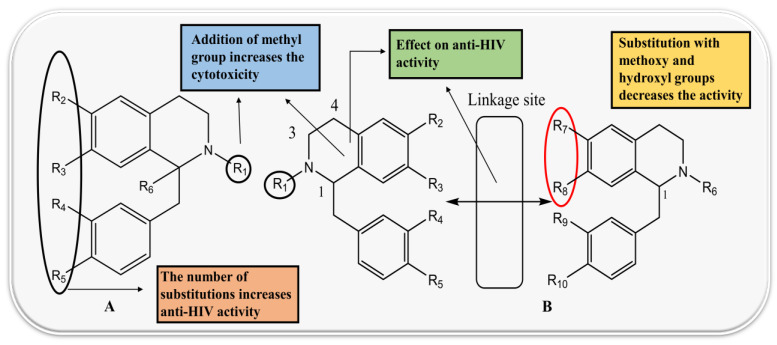
Structure–activity relationship of benzylisoquinoline (**A**) and bisbenzyl-isoquinoline alkaloids (**B**).

The BBI alkaloids category seems to have an important role in the treatment and prevention of SARS-CoV-2 and other viral infections. Some alkaloids categorized under this division were evaluated for various viral targets and produced effective results. The structure–activity relationship of this category is depicted in Figure 6.

### 4.5. Ipecac Alkaloids

A natural alkaloid, emetine present in the plant *Psychotria ipecacuanha*e (Brot.) Standl. has been reported against HIV with 80% inhibition [104]. Emetine showed inhibition of Zika virus nonstructural protein-5 (NS5) polymerase activity and ebolavirus (EBOV) with effective IC_50_ values. They disrupted the entry of lysosomal function and exerted anti-viral action [150]. The semi-synthetic derivative of emetine known as emetine dihydrochloride hydrate is found to be effective against SARS and MERS with EC_50_ values of 0.051 and 0.014 respectively [103]. A study identified four alkaloids including emetine as potential antiviral agents against echovirus (EV-1), and herpes simplex virus (HSV-2) on retinal pigment epithelial (RPE) cells [151].

The salts of ipecac alkaloids such as psychotrine dihydrogen oxalate and o-methylpsychotrine sulfate heptahydrate also exhibited potential inhibitory activity towards reverse transcriptase of HIV [152]. Some other alkaloids such as o-methylpsychotrine sulfate and papaverine also demonstrated anti-HIV activity [153]. One study investigated the effect of seven compounds including lycorine and emetine on HCoV-OC43 and MERS-CoV. Findings stated that lycorine suppressed the viral load in the BALB mice model and represented anti-HCoV-OC43 potential. However, emetine was found effective against MERS-CoV [111].

### 4.6. Naphythylisoquinoline Alkaloids

Michellamines alkaloids from *Ancistrocladus congolensis* J. were identified for their anti-viral action and found effective against HIV-1 and HIV-2 [104,105,154,155,156]. A study indicated that michellamine A2, A3, A4, and michellamine B showed IC_50_ values of 29.6, 15.2, 35.9, and 20.4 µM respectively, and thus exerted anti-HIV action [106]. Another study isolated korupensamine E along with michellamine B, D, E, and F from the same plant and was investigated for *in vitro* HIV activity. Results confirmed that all michellamine possessed antiviral activity against HIV with EC_50_ values in the range of 17–188 µM [157]. A bis-benzylisoquinoline alkaloid neferine and their desmethyl and didesmethyl analogues such as isoliensinine and liensinine also showed effective activity against HIV [89].

### 4.7. Pavine Alkaloids

A pavine alkaloid (−)-thalimonine reduces the apoptosis caused by viral proteins and possessed anti-HSV action on wild-type cells [107]. However, Cherylline inhibits DENV and Zika virus replication with an EC_50_ value of 8.8 µM and 20 µM [158].

### 4.8. Morphinan and Promorphinan Alkaloids

The skeleton of morphinan alkaloids is very similar to aporphine alkaloids except for one extra ring closure [41]. Morphine inhibits interferon (IFN) in peripheral blood mononuclear cells (PBMC) cells and thus demonstrated anti-HIV activity [159]. A study includes the anti-HSV activity of multiple morphinan alkaloids such as FK-3000, cephakicine, sinoacutine, cephasamine, cephamonine, sinomenine, 14-episinomenine, and tannagine. Among all, the two main morphinan alkaloids namely FK-3000 and cephakicine demonstrated effective results against HSV-1 with IC_50_ value of 7.8 and 44.4 µg/mL respectively. However, in addition, FK-3000 also showed its notable activity against HSV-1 TK^-^ and HSV-2 demonstrating 9.9 and 8.7 IC_50_ values respectively [93].

### 4.9. Benzophenanthridines

The higher plants mainly consist of benzophenanthridine isoquinoline compounds exhibit a wide range of pharmacological activities. A study investigated 2000 synthetic and natural compound which include sanguinarine as a HIV-protease inhibitor with IC_50_ value of 4.3 µg/mL [160]. An anti-HIV-1 and HIV-2 reverse transcriptase (RT) study included fangronine chloride and nitidine chloride to determine the antiviral potential. The IC_50_ value of fangronine chloride (8.5 µg/mL) and nitidine chloride (7.4 µg/mL) suggest the efficacy of these two compounds against HIV-RT. Similar results were shown in the case of HIV-2 RT where fangronine chloride displayed a 9.5 µg/mL IC_50_ value, whereas, nitidine chloride displayed a 7.1 µg/mL IC_50_ value [109]. Anti-HCV and PI-3 (para-influenza) activity of norsanguinarine displayed moderate action against HCV and PI-3 with minimum inhibitory concentration (MIC) range from 16 to 32 µg/mL [76].

## 5. Inflammation Inhibition

Anti-inflammatory phytochemicals might be viable therapeutic candidates against various viruses, including SARS-CoV2, as they have direct antiviral effects and can alleviate the status of inflammatory diseases [22,23]. Moreover, it is evident from recent reports that severe COVID-19 patients cause elevation of inflammatory pro-markers which eventually lead to multiorgan failure [161]. Thus, researchers are now focusing on natural products such as medicinal plants that possess anti-inflammatory as well as anti-viral potential. Tetrandrine from the category of bis-benzylisoquinoline alkaloids of isoquinoline alkaloids has been reported for immunomodulation and anti-inflammatory activities. It has anti-inflammatory action against croton oil-induced ear edema in mice and demonstrates 95% inhibition at 12.5 µM [162]. This isoquinoline alkaloid also showed inhibition against proinflammatory mediators of cytokines, iNOS, and cyclooxygenase (COX-2) in human monocytic cells [163]. In vitro lipopolysaccharide assay suggested the potential of tetrandrine to inhibit TNF-α, IL-6, and NO release in lipopolysaccharide (LPS)-induced microglial activation [164]. Similarly, in vivo studies performed on BBR, which is another isoquinoline alkaloid at the dose of 50 mg/kg, indicated a reduction of IL-6, IL-10, IL-1β, and IFN-γ caused by thioacetamide injection [165]. Noscapine is also found to be responsible for showing inhibitory action against cytokine storm in COVID-19 [166]. Recently, an in vitro study indicated that litcubanine, an isoquinoline alkaloid greatly reduced the activation of inflammatory macrophages by LPS via the NF-κB pathway, which would reduce the levels of inflammatory mediators such iNOS, TNF-α, and IL-1β [167].

## 6. Clinical Findings

The efficacy of isoquinoline and related alkaloids in various scientific reports including in silico, in vitro, and in vivo evaluation justified a slew of human clinical trials to assess the safety, pharmacokinetics, and pharmacodynamic effectiveness in a variety of viral pathologies. Few recent clinical trials also justify the role of isoquinoline and related alkaloids in viral infections. For example, clinicians investigated the effects of BBR (NCT04479202) on intestinal function, serum concentrations of inflammatory biomarkers, and organ function in severe SARS-CoV-2-infected patients in a prospective randomized controlled clinical trial supported by the Chinese medical association. An herbal formulation of COVIDEX also contains BBR (NCT05228626) targeting SARS-CoV-2 in a clinical trial sponsored by Makerere University in Uganda. Tetrandrine (NCT04308317) in combination with standard drug improves prognosis and reduces the incidence of pulmonary fibrosis during rehabilitation in SARS-CoV-2 patients. Even though there are only a few clinical trials (enlisted in Table 3) in the literature that include the use of isoquinoline and related alkaloids with various viral infections, these moieties are expected to have a greater impact on viral load, making these molecules the alternative option for improving the limited clinical efficacy and expanding the field of use to other viral pathologies.

## 7. Conclusions and Future Perspective

The emerging variants of powerful viruses have made the task of clinical practitioners and scientists more challenging to find an effective solution against viral infections. However, naturally occurring isoquinoline and related alkaloids could be an alternative option for treating these rapidly mutating viruses. From research findings, it can be concluded that these alkaloids exert their antiviral action majorly by interfering with the signaling pathways such as NF-κB, and MEK/ERK that eventually restricts the entry and replication of the virus. Furthermore, inhibition of Ca^2+^-mediated fusion and SandN protein expression could also facilitate the antiviral actions. Isoquinoline and their related alkaloids also resist pro-inflammatory markers such as IL-6, IL-10, and IL-1β and exert anti-inflammatory action. Additionally, an increase in CD8+ cells and IFN-γ production by some of the isoquinoline alkaloids represent immunomodulatory effects.

The structure–activity relationship has clearly shown that the quaternary nitrogen atom is essential for the antiviral activity of protoberberine and benzophenanthridine alkaloids. However, substitution with a methyl group at benzyltetrahydroisoquinoline and BBI moieties potentiate the antiviral action. From the in vitro and in vivo studies, it is concluded that isoquinoline and related alkaloids including berbamine, CEP, tetrandrine, neferine and lycorine from the BBI category of isoquinoline alkaloids represent broad-spectrum activities against HSV, HIV, SARS-CoV, and SARS-CoV-2 infection. Thus, BBIalkaloids should be further structurally explored for the treatment of newly emerged viral strains.

## Figures and Tables

**Table 1:** In silico binding affinities of isoquinoline and related alkaloids against SARS-CoV-2.

Sr. No.	Alkaloid	Isoquinoline and Related Alkaloid	Structure	Target	Binding Score/Affinity	Docking Software	References
**1.**	Thebaine	aporphine alkaloid	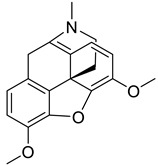	ACE2, S-protein	−100.77 and −103.58 kcal/mol	Molegro Virtual Docker 3.0.0	[46]
**2.**	Berberine	Protoberberine alkaloid	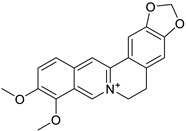	ACE2, S-protein, 3CL^pro^	−97.54, −99.93 and −7.3 kcal/mol	Auto Dock; Auto Dock Vina	[46,47]
**3.**	Lycorine	Tetrahydroisoquinolines	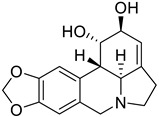	S1 subunit; SARS-CoV-2	−86.92 kcal/mol	Discovery Studio 2.5	[42]
**4.**	Tylophorine	phenanthraindolizidine alkaloid	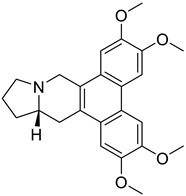	S1 subunit; SARS-CoV-2	−89.77 kcal/mol	Discovery Studio 2.5	[42]
**5.**	Tetrandrine	bis-benzylisoquinoline alkaloid	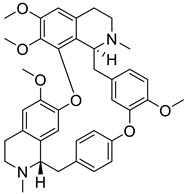	S1 subunit; SARS-CoV-2	−72.96 kcal/mol	Discovery Studio 2.5	[42]
**6.**	Fangchinoline	bis-benzylisoquinoline	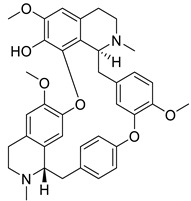	S1 subunit; SARS-CoV-2	−92.66 kcal/mol	Discovery Studio 2.5	[42]
**7.**	Berbamine	bis-benzylisoquinoline	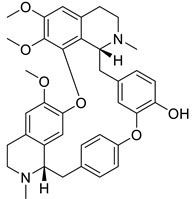	M^pro^	−20.79 kcal/mol	PyRx	[48]
**8.**	Noscapine	phthalideisoquinoline alkaloid	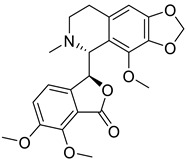	M^pro^	−292.42 kJ/mol	Hex 8.0	[49]
**9.**	Palmatine	protoberberine	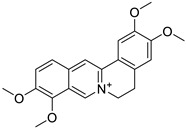	M^pro^	>8 kcal/mol	Swiss Dock; EADock DSS	[50]
**10.**	Morphine	aporphine alkaloid	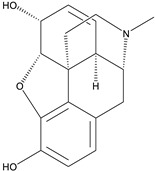	M^pro^	>−6.0 kcal/mol	AutoDock Vina v.1.0.2.	[51]
**11.**	Codeine	aporphine alkaloid	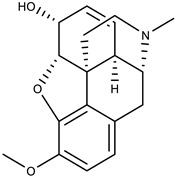	M^pro^	>−6.0 kcal/mol	AutoDock Vina v.1.0.2.	[51]
**12.**	Cepharanthine	biscoclaurine alkaloid	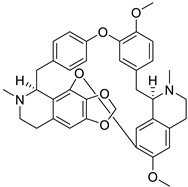	3CL^pro^, S1-RBD, and TMPRSS-2	−8.5, −106.74, −7.4 kcal/mol	Auto Dock Vina v.1.0.2; Discovery Studio 2.5	[42,52]
**13.**	Cassiarin D	isoquinoline alkaloid	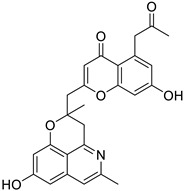	RdRP	−150.7 kJ/mol	Molegro Virtual Docker (version 6.0, Molegro ApS, Aarhus, Denmark))	[53]
**14.**	Coptisine	protoberberine	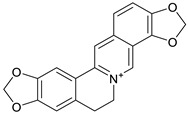	M^pro^, N3-M^pro^	−9.15 and −8.17 kcal/mol	Autodock (version 4.2)	[54]
**15.**	Emetine	Ipecac alkaloids	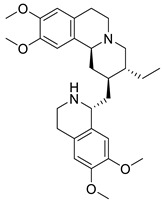	M^pro^	−10.17 kcal/mol	Autodock version 4.2.6	[55]

**Table 2:** In vitro activities of isoquinoline and related alkaloids against several viral infections.

Sr. No.	Active Compound	Cell Lines/model	IC_50_/CC_50_ Value	EC_50_/ED_50_ Value	Mechanism/Activity	Antiviral Activity	References
Protoberberine Alkaloids							
1.	Berberine	HEC-1-A cells HEK293T	165.7 µM	-	suppression of HSV-induced NF-κB activation, IκB-α degradation and p65 nuclear translocation	HSV	[60]
		J774A.1 cells	-	-	inhibition of HIV-PI induced TNF-α and IL-6 in macrophages	HIV	[61]
		Calu-3 cells	-	-	increases CD8^+^ cells and IFN-γ to produce immunomodulatory activity	SARS-CoV and SARS-CoV-2	[62]
		Vero E6 cells	10.58 µΜ	-	drug antagonism was seen (remdesivir + BBR)	SARS-CoV-2	[63]
		Vero E6 cell	-	16.70 μM	suppression of SARS-CoV-2 target proteins	SARS-CoV-2	[64]
		VeroE6 cells and nasal epithelial cells	-	9.1 µM	inhibition of SARS-CoV-2 replication	SARS-CoV-2	[65]
		RAW 264.7 macrophage and A549 cells	0.44 μM	-	inhibition of viral growth of influenza and suppresses production of TNF-α and PGE2	H1N1	[66]
		human osteosarcoma cell (HOS)	-	12.2 µM	inhibits MAPK/PI3K-AKt signaling pathways	Chikungunya virus	[67]
		A549 cells and in vivo (mice)	-	-	BBR suppresses TLR7, MyD88, and NF-κB to resist viral replication in mice	H1N1	[68,69]
		HFF, HELF, and NIH 3T3	-	2.65 µM	inhibition of DNA polymerase and immediate-early (IE-3) proteins	HCMV	[70]
		A549, LET1, HAE, and MDCK	17 µM, 4 µM, 16 µM, and 52 µM	-	interfere MEK/ERK pathway to inhibit export of the viral ribonucleoprotein	H1N1	[68]
2.	Saxicolalines A	Hep G 2.2.15 cell line	2.19 and >2.81 against HBsAg ^b*^ and HBeAg ^c*^ respectively	-	-	HBV	[71]
3.	N-methylnarceimicine	Hep G 2.2.15 cell line	1.24 µM and 1.86 µM against HBsAg ^b^ and HBeAg ^c^ respectively	-	-	HBV	[71]
Aporphine Alkaloids							
1.	Roemerine	H9 cells	-	0.84 μg/mL	inhibition of HIV replication by 50%	HIV-1	[72]
2.	Nuciferine			0.8 μg/mL			
3.	Nornuciferine			<0.8 μg/mL			
4.	Boldine	PMBMCs, CEM-GXR cells, Vero cells	207.7, 250 µM	50.2 µM	inhibit HIV-1 replication, reduction of the viral titer	HIV-1, poliovirus	[73,74]
5.	Dasymaroine A	C8166 cell line	-	1.93 to 9.70 µM	reduce viral replication by 50%	HIV-1	[75]
6.	Dasymaroine B						
7.	Laurolitsine	Vero cells	95 µM	62 µM	reduction of the viral titer	Poliovirus	[74]
8.	Isoboldine HCL		217 µM	−15 µM			
9.	Glaucine fumarate		142 µM	9 µM			
10.	N-acetylnorglaucine		342 µM	50 µM			
11.	N-methyllaurotetanine		250 µM	15 µM			
12.	laurotetanine, HCl		165 µM	31 µM			
13.	(+)-bulbocapnine	MDBK and Vero cell lines	-	-	-	PI-3	[76]
14.	Pachystaudine	Vero cells	68 µM	−31 µM	reduction of the viral titer and viral replication	Poliovirus and HSV	[74,77]
15.	Oxostephanine		28 µM	-			
16.	Oliverine HCl	Vero cells	9 µM	-	reduction of the viral titer	Poliovirus	[74]
Benzylisoquinoline and Benzyltetrahydroisoquinoline Alkaloids							
1.	(+)-1(R)-coclaurine	H9 cells	>100 µg/mL	0.8 µg/mL	inhibition of HIV replication by 50%	HIV-1	[72]
2.	(−)-1(R)-N-methylcoclaurinre		1.45 µg/mL	<0.8 µg/mL			
3.	(−)-1(S)-norcoclaurine		20 µg/mL	<0.8 µg/mL			
4.	Armepavine		1.77 µg/mL	<0.8 µg/mL			
5.	Lotusine		>100 µg/mL	20.7 µg/mL			
6.	Papaverine	Vero E6, Caco-2, and BHK-21 cells	1.1 µM	-	reduce SARS-CoV-2 cytotoxic effect	SARS-CoV-2	[78]
7.	Papaverine hydrochloride	MT4 cells		5.8 µM	inhibition of viral replication	HIV	[79]
Bis-benzylisoquinoline Alkaloids (BBI)							
1.	Aromoline	MT-4 cells	-	IC_100_ 31.3µg/mL	cytopathic effect of HIV is inhibited	HIV-1	[80]
2.	Cycleanine	HCT-116	-	1.83 µg/mL	-	HIV-2	[81]
3.	Fangchinoline	MT-4, PM1, and 293T cells	-	0.8 to 1.7 µM	interfering with gp160 proteolytic cleavage thus inhibit viral replication	HIV-1	[82]
		MRC-5 fibroblast lung cell line	1.01 µM	-	inhibition of S and N protein expression as well as HCoV-OC43 replication	HCoV-OC43	[83]
4.	Tetrandrine	BALB	-	-	IL-6 inhibition in the corneal inflammation	HSV	[84]
		HEK 293T cells expressing ACE2	-	>10 µM	suppress viral entry by inhibiting Ca^2+^-mediated fusion	SARS-CoV-2	[85]
		MRC-5 cells	0.33 μmol/L	-	two-pore channel 2 inhibition	SARS-CoV-2	[86,87]
		MRC-5 fibroblast lung cell line	0.33 µM	-	inhibition of S and N protein expression as well as HCoV-OC43 replication	HCoV-OC43	[83]
5.	Neferine	HEK293/hACE2 and HuH7 cell lines	-	0.13–0.41 µΜ	blockage of viral entry by inhibiting Ca^2+^-mediated fusion	HCOV OC43	[88]
		Dog hepatic microsomes	-	-	N-demethylation and O-demethylation	HIV	[89]
		HEK 293T cells expressing ACE2	-	>10 µM	suppress viral entry by inhibiting Ca^2+−^-mediated fusion	SARS-CoV-2	[85]
		Huh7, HEK293/hACE2 cell lines	-	0.36 µM	blockage of Ca^2+^ dependent membrane fusion	SARS-CoV-2	[88]
6.	Berbamine	Hela cells, A549 cells	-	JEV (1.62 µM) ZIKV (2.17 µM)	inhibiting Ca^2+^ channel transient receptor potential membrane channel mucolipin (TRPML)	JEV andZIKV	[90]
		Huh7-cells	-	~2.4 μM	inhibition of Ca^2+^ influx and TRPMLs	SARS-CoV-2	[91]
		Vero E6 cells	5.79 µM	0.94 µM	inhibition to the 2-E channel	SARS-CoV-2	[92]
		Vero cells	16.3–24.9 µg/mL	-	-	HSV-1 TK and HSV-2	[93]
7.	Cepharanthine	U1 and ACH-2	-	0.016 µg/mL	inhibition of NF-κB pathway	HIV-1	[94]
		U937 cells	4.6 µg/mL	-	inhibition of inflammatory cytokines	HIV-1	[95]
		PHA-blast cells from PBMC, HEK 293T, MOLT4 cells	CC_50_—10.0 µg/mL	-	reduce plasma membrane fluidity to block the NF-κB pathway and HIV-1 entrance	HIV-1	[96]
		U1 cells	-	0.0041 µg/mL	inhibition of NF-κB pathway	HIV-1	[94,97]
		Vero cells and Hela cells	0.835 μg/mL	-	inhibition of NF-κB	HSV	[98,99]
		MRC-5 fibroblast lung cell line	0.83 µM	-	inhibition of viral S and N protein expression as well as HCoV-OC43 replication	HCoV-OC43	[83]
		Vero E6 cells	6.0 µg/mL–9.5 µg/mL	-	-	SARS-CoV	[100]
		HEK 293T cells expressing ACE2	-	>10 µM	suppression of viral entry through inhibition of Ca^2+^-mediated fusion	SARS-CoV-2	[85]
		Vero E6 cells	0.91 µM	-	combination of NFV and CEP reduces viral RNA levels	SARS-CoV-2	[101]
8.	di-O-acetylsinococuline (FK-3000)	MT-4 cells	CC_0_ 15.6 µg/mL	-	inhibition of HIV cytopathic effect	HIV-1	[80]
9.	12-O-ethylpiperazinyl cepharanoline	U1 cells	-	0.028 µg/mL	inhibition of NF-κB pathway	HIV-1	[97]
10.	Homoaromoline	Vero cells	16.3–24.9 µg/mL	-	-	HSV-1 TK and HSV-2	[102]
11.	Isotetrandrine						
12.	Thalrugosine						
13.	Obamegine						
14.	Hernandezine	HEK 293T cells	-	>10 µM	inhibition of Ca^2+^-mediated fusion	SARS-CoV-2	[85]
Ipecac Alkaloids							
1.	Emetine	HEK293 cells	93–52.9 nM	-	inhibit MERS-CoV entry	MERS-CoV	[103]
		SNB-19 cells	29.8 nM	-			
		Vero E6 cells	8.74 nM	-			
		BHK21 cells	0.30 µM				
		Vero E6, Caco-2, and BHK-21 cells	0.52 µM	-	inhibit cytotoxic effect of SARS-CoV-2	SARS-CoV-2	[78]
2.	Emetine dihydrochloride hydrate	Vero E6 cells	-	0.051 µM	inhibition of viral replication -	SARS	[103]
				0.014 µM		MERS	
Naphthylisoquinoline Alkaloids							
1.	Michellamine A	MT-2 target, CEM-SS cells	-	EC_50_~20 µM	inhibition of viral replication	HIV-1 and HIV-2	[104]
2.	Michellamine B	MT-2 target, CEM-SS cells	-	EC_50_~20 µM	inhibition of viral replication	HIV-1 and HIV-2	[104,105]
		CEM-SS and MT-2	-	18 µg/mL	inhibition of viral replication	HIV-1 and HIV-2	[104]
		H9 Cells	20.4 µM	-			
3.	Michellamine A2	H9 Cells	29.6 µM	-	inhibition of HIV replication	HIV-1	[106]
4.	Michellamine A3	H9 Cells	15.2 µM	-		HIV-1	[106]
5.	Michellamine A4	H9 Cells	35.9 µM	-		HIV-1	[106]
Pavine Alkaloid							
1.	(−)-Thalimonine	MDBK cells	-	-	restoration of apoptosis during viral replication	HSV	[107]
Morphinan and Promorphinan							
1.	FK-3000	Vero cells	7.8 µg/mL	-	reduction in plaque formation	HSV	[93]
2.	Cephakicine		44.4 µg/mL				
3.	Sinoacutine		>50 µg/mL				
4.	Cephasamine						
5.	Cephamonine						
6.	Sinomenine						
7.	14-episinomenine						
8.	Tannagine						
Benzophenanthridines							
1.	Fagaronine	Vero cells	CC_50_ > 0.3 mM	-	inhibition of topoisomerases I and II	HSV	[108]
2.	Fagaronine chloride	HIV-1 and HIV-2 RT assays	IC_50_—9.5 µg/mL	-	inhibit RNA and DNA polymerizing enzymes	HIV-2 RT	[109]
3.	Nitidine chloride		IC_50_—7.1 µg/mL	-			
4.	Sanguinarine	HTS (*E. coli*-based assay)	IC_50_—12.82 µM	-	inhibits proteolytic activity	HIV-1	[110]
5.	Chelerythrine		IC_50_—38.71 µM				
6.	Lycorine	BHK21 cells	-	0.15 µM	viral load suppression	HCoV-OC43	[111]

*HBsAg ^b^: hepatitis B virus surface antigen and HBeAg^c^: hepatitis B virus e antigen

**Table 3:** Clinical trials of isoquinoline alkaloids against viral infections.

Sr. No.	Title	Phase	Viral Infection	Study	Completion of Study	Origin	NCT Number
1	The Effect of Berberine on Intestinal Function and Inflammatory Mediators in Severe Patients with Covid-19 (BOIFIM)	Phase 4	COVID-19	Interventional	April 2020	Chinese medical association	NCT04479202
2	Effect of Berberine on Metabolic Syndrome, Efficacy and Safety in Combination with Antiretroviral Therapy in PLWH. (BERMESyH)	Phase 3	HIV-1	Interventional	July 2022	Hospital Civil de Guadalajara	NCT04860063
3	Tetrandrine Tablets Used in the Treatment of COVID-19 (TT-NPC)	Phase 4	COVID-19	Interventional	May 2021	Henan Provincial People’s Hospital	NCT04308317
4	Study of Oral High/Low-dose Cepharanthine Compared with Placebo in Non-Hospitalized Adults with COVID-19	Phase 2	COVID-19	Interventional	August 2022	Hai Li, Shanghai Jiao Tong University School of Medicine	NCT05398705
5	Measurement of the Efficacy of MORPHINE in the Early Management of Dyspnea in COVID-19 Positive Patients (CODYS)	NA	COVID-19	Observational	February 2021	Hospices Civils de Lyon	NCT04522037
6	Safety and Efficacy of COVIDEX™ Therapy in Management of Adult COVID-19 Patients in Uganda. (COT)	Phase 2	COVID-19	Interventional	December 2022	College of Health Sciences, Makerere University	NCT05228626

## Data Availability

Not applicable.
